# 非小细胞肺癌中SLC22A18的表达及其临床意义

**DOI:** 10.3779/j.issn.1009-3419.2012.01.04

**Published:** 2012-01-20

**Authors:** 鸣 雷, 庆书 程, 亚超 赵, 涛 刘, 雪娇 王, 迎春 邓, 菁 杨, 志培 张

**Affiliations:** 710038 西安，第四军医大学唐都医院胸外科 Department of Thoracic Surgery, Tangdu Hospital, the Fourth Military Medical University, Xi' an 710038, China

**Keywords:** 肺肿瘤, SLC22A18, 免疫组织化学, Lung neoplasms, SLC22A18, Immunohistochemistry

## Abstract

**背景与目的:**

已有的研究证明：多药耐药（multidrug resistance, MDR）是肺癌化疗失败的主要原因，研究MDR的产生机制对于提高肺癌的化疗疗效有着重要的临床意义。*SLC22A18*基因编码蛋白与跨膜转运体相似，影响药物敏感性、细胞代谢和生长，可能在肺癌MDR的产生中发挥一定作用。本研究旨在检测SLC22A18在非小细胞肺癌（non-small cell lung cancer, NSCLC）及相应正常组织中的表达，并分析其与组织学类型、分级和TNM分期的关系。

**方法:**

应用免疫组化EnVinsion法检测SLC22A18在96例NSCLC及正常组织中的表达，结果用统计学软件SPSS 17.0进行分析。

**结果:**

SLC22A18主要定位于胞膜和胞质中。SLC22A18在NSCLC中的表达高于正常组织，差异明显（*P*＜0.01），鳞癌、腺癌阳性率分别为68.0%和78.2%，差异性有统计学意义（*P*＜0.05）。鳞癌、腺癌不同病理分级、TNM分期间SLC22A18表达差异性均有统计学意义，癌组织分化越差、分期越晚，SLC22A18表达越高（*P*＜0.05）。

**结论:**

SLC22A18在NSCLC组织中高表达，表达的高低与组织学类型、分级、TNM分期有关，本研究为进一步探讨SLC22A18在肿瘤中的表达及可能的耐药作用提供了实验依据。

## Abstract (en)

**Background and objective:**

It has been proven that multidrug resistance (MDR) is the main cause of chemotherapy failure in lung cancer. Research on emergence mechanisms of MDR has great clinical significance in improving the curative efficiency of lung cancer chemotherapy. Proteins encoded by the *SLC22A18* gene, which is similar to the transmembrane transporter, may influence the sensitivity of chemotherapeutics as well as the metabolism and growth of cells. In addition, these proteins probably have some effect on the development of lung cancer MDR. The aim of the present study is to investigate the expression of SLC22A18 protein in non-small cell lung cancer (NSCLC) as well as in corresponding normal lung tissue. Furthermore, the relationship between SLC22A18 expression and pathological grade and TNM stage is analyzed.

**Methods:**

The expression of SLC22A18 was detected by EnVinsion in 96 cases with NSCLC and in corresponding normal lung tissue. Statistical analysis was performed using SPSS 17.0 statistical software.

**Results:**

SLC22A18 was mainly located in cell membrane and cytoplasm. The expression level of SLC22A18 in NSCLC was significantly higher than that in normal tissue (*P* < 0.01). The positive rates in squamous cell lung cancer and lung adenocarcinoma were 68% and 78.2%, respectively (*P* < 0.05). Moreover, the higher expression of SLC22A18 was associated with lower histological grade and later TNM stage (*P* < 0.05).

**Conclusion:**

SLC22A18 protein is overexpressed in NSCLC, and its expression is correlated with pathological grade and TNM stage. These findings provide the experimental basis for investigating the role of tumor and chemoresistance.

肺癌是最常见的恶性肿瘤之一，在我国其发病率及死亡率呈逐年上升趋势，是国内首位恶性肿瘤死亡原因^[1, 2]^。目前化疗仍是肺癌综合治疗的主要手段之一，在非小细胞肺癌（non-small cell lung cancer, NSCLC）的治疗中至关重要，研究^[3]^表明多药耐药（multidrug resistance, MDR）是导致NSCLC化疗失败的主要原因。*SLC22A18*（Solute Carrier Family 22, member 18）是近年来发现的一个新的父系印迹基因，与肾母细胞瘤、横纹肌肉瘤、肝母细胞瘤等多种肿瘤的发生有关^[4]^。该基因编码蛋白与跨膜转运体相似，影响药物敏感性、细胞代谢和生长^[5]^，研究^[6]^表明其在细菌中的表达与细菌耐药性有关，提示其在哺乳动物细胞中可能具有相似的作用，可能在肺癌MDR的产生中发挥一定作用。本实验通过免疫组化方法检测SLC22A18在NSCLC组织中的表达并分析其与NSCLC生物学、临床特征的相关性，为进一步研究SLC22A18与肿瘤耐药的关系提供实验基础。

## 1 材料与方法

### 1.1 标本来源

收集唐都医院胸外科2005年10月-2006年6月手术切除的NSCLC新鲜标本96例。男61例，女35例。年龄34岁-73岁，中位年龄49岁。其中腺癌46例，鳞癌50例。所有患者术前均未接受过放、化疗，术后即刻切取标本，常规中性福尔马林固定石蜡包埋，连续切片（厚度4 μm），用于免疫组化染色检测相关蛋白的表达。组织病理分型、分级由病理科医师诊断，术后进行临床分型。

### 1.2 试剂

SLC22A18兔抗人多克隆抗体购自Abcam公司，抗兔二抗及免疫组化试剂盒购自西安壮志生物技术公司。

### 1.3 方法

免疫组化染色采用两步法（EnVinsion^TM^），步骤如下：切片脱蜡至水，尿素消化，3%过氧化氢封闭，用柠檬酸缓冲液进行微波修复，冷却，兔血清封闭，加一抗（1:50）4 ℃过夜。从冰箱取出，37 ℃复温，自来水冲洗抗体，加入抗兔二抗（EnVinsion），37 ℃恒温反应（中间各步均用PBS冲洗），DAB显色，显微镜观察终止显色。苏木精轻微复染，脱水透明封片，在光镜下观察。对照组设正常组织和空白（以PBS代替一抗）对照。

### 1.4 判定标准

SLC22A18以细胞膜及胞质中出现棕黄色颗粒为阳性细胞，每例均随机观察5个高倍视野（×400），判断结果按照二级计分法，阳性细胞数目＜5%为0分，5%-25%为1分，26%-50%为2分，51%-75%为3分，＞75%为4分。染色强度分类：淡黄色为1分，黄色及深黄色为2分，褐色及深褐色为3分。将细胞阳性率与染色强度两者积分相乘：0分为阴性（-），1分-4分为弱阳性（+），5分-8分为中度阳性（++），9分-12分为强阳性（+++）。

### 1.5 统计学方法

使用SPSS 17.0软件进行统计学处理，非小细胞肺癌和正常组织、鳞癌和腺癌的比较分析采用两组等级资料*Mann*-*Whitney U*秩和检验，鳞癌和腺癌临床分型比较分析采用*Kruskal*-*Wallis*秩和检验，以*P*＜0.05为差异具有统计学意义。

## 2 结果

免疫组化结果显示，SLC22A18阳性表达主要定位于胞膜上和胞质中（图 1A，图 1B），SLC22A18在肺鳞癌空白对照及正常肺组织中阴性表达（图 1C，图 1D）。非小细胞肺癌和正常组织中的阳性表达率分别为72.9%、52.1%，差异明显（*P*＜0.001）（表 1）。

**1:**
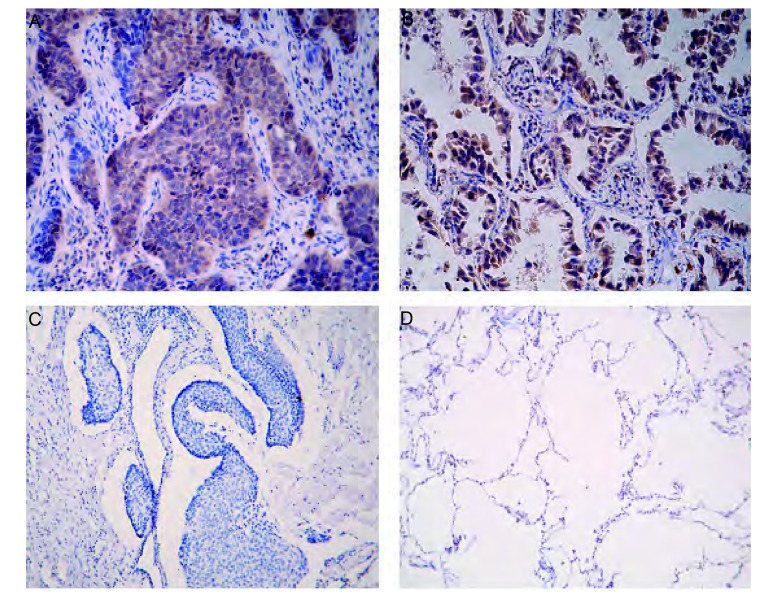
SLC22A18在NSCLC及正常肺组织中的表达。A：SLC22A18在肺鳞癌中的表达（×200）；B：SLC22A18在肺腺癌中的表达（×200）；C：SLC22A18在肺鳞癌空白对照（×100）；D：SLC22A18在正常肺组织中的阴性表达（×100）。 Expression of SLC22A18 in NSCLC and normal lung tissue. A: Expression of SLC22A18 in lung squamous cell carcinoma (×200); B: Expression of SLC22A18 in lung adenocarcinoma (×200); C: Blank control in lung squamous cell carcinoma (×100); D: Expression of SLC22A18 in normal lung tissue (×100).

**1:** 正常组织、癌组织间及鳞癌、腺癌间SLC22A18表达差异 Comparison of SLC22A18 protein between NSCLC and normal lung tissue

Group	*n*	SLC22A18			Positive (%)	*U*	*P*
		(-)	(+)	(++)			
Normal	96	46	37	13	52.1	3, 303	< 0.001
Carcinoma	96	26	38	32	72.9		
Squamous cell carcinoma	50	16	24	10	68.0	838	0.015
Adenocarcinoma	46	10	14	22	78.2		

鳞癌中SLC22A18的总体阳性表达率为68.0%，腺癌为78.2%，差异具有统计学意义（*U*=838, *P*=0.015）（表 1）；其中Ⅱ级、Ⅲ级鳞癌阳性率分别为54.2%、80.8%，差异明显（*P*=0.041），中分化、低分化腺癌阳性率分别为69.6%、86.9%，差异明显（*P*=0.007）（表 2）；鳞癌、腺癌不同TNM分期之间SLC22A18的表达差异均有统计学意义（*P*值分别为0.044、0.034）（表 3）。

**2:** 鳞癌、腺癌不同病理分级间SLC22A18表达差异 Expression of SLC22A18 in squamous and adenocarcinoma cell carcinoma with different pathological grade

Pathological grade		*n*	SLC22A18			Positive (%)	*U*	*P*
			(-)	(+)	(++)			
Squamous cell carcinoma	Moderate	24	11	10	3	54.2	215	0.041
	Poor	26	5	14	7	80.8		
Adenocarcinoma	Moderate	23	7	10	6	69.6	151	0.007
	Poor	23	3	4	16	86.9		

**3:** 鳞癌、腺癌不同TNM分期间SLC22A18表达差异 Expression of SLC22A18 in squamous and adenocarcinoma cell carcinoma with different TNM stage

TNM stage		*n*	SLC22A18			*χ*^2^	*P*
			(-)	(+)	(++)		
Squamous cell carcinoma	Ⅰ	7	2	5	0	6.26	0.044
	Ⅱ	33	13	15	5		
	Ⅲ	10	1	4	5		
Adenocarcinoma	Ⅰ	5	2	2	1	6.74	0.034
	Ⅱ	29	8	9	12		
	Ⅲ	12	0	3	9		

## 3 讨论

肺癌目前采用手术、化疗、放疗等多学科综合治疗，多数患者确诊时已属晚期，失去了手术机会，因而化疗在肺癌治疗中占据重要的地位，但肿瘤的多药耐药常常导致NSCLC化疗失败，其中药物转运蛋白活性增强是其重要原因之一。因此，肿瘤的耐药基因及耐药蛋白仍是肿瘤治疗的研究热点。

基因*SLC22A18*位于人染色体11p15.5，转录方向从端粒到中心粒，有11个外显子，cDNA全长1.5 kb，蛋白分子量43 kDa。研究^[7]^表明，SLC22A18编码的蛋白有10个跨膜域，与跨膜转运体蛋白和有机阳离子转运体（organic cation transporters, OCT）具有一定同源性。SLC22A18与多药耐药基因1（multidrug resistance 1, MDR1）及乳癌耐药蛋白（breast cancer resistance protein, BCRP）^[8, 9]^同属于跨膜转运体蛋白家族，故推测其可能在肿瘤细胞中具有外排化疗药物从而产生多药耐药的作用。已有研究^[10]^证明SLC22A18过表达后，乳腺癌细胞耐药性有所增强，但关于非小细胞肺癌中SLC22A18对化疗药物敏感性影响的具体研究目前还鲜见报道。

*SLC22A18*基因在胎儿肝脏、肾脏以及多种成体心脏、肝脏、肾脏、脾脏、胸腺、前列腺、睾丸和小肠等组织中表达^[5, 11]^。在肿瘤细胞中表达的研究较少，尤其是在NSCLC中的表达未见报道。本实验结果显示SLC22A18在NSCLC中的表达（72.9%）高于相应正常组织（52.1%），两者差异具有统计学意义（*P*＜0.001），提示SLC22A18在NSCLC的发生、发展中可能起一定的作用，并且可能对肺癌细胞的化学敏感性产生影响。肺鳞癌、腺癌中SLC22A18都有较高的表达率（68%, 78.2%），尤其腺癌中表达更高，两者差异具有统计学意义（*P*＜0.05），提示SLC22A18的表达可能是腺癌多药耐药发生率高于鳞癌的机制之一，原因可能是在腺癌中*SLC22A18*基因在转录、表达调控、氨基酸组装及调控水平的差异，其具体机制有待进一步研究。另外我们发现鳞癌、腺癌不同病理分级、不同TNM分期与SLC22A18的表达具有相关性，癌组织分化越差、分期越晚，表达越高（*P*＜0.05），说明其表达高低与肿瘤的恶性生物学行为有关，并且与临床上NSCLC化疗晚期患者较早期患者相对不敏感相吻合，提示SLC22A18可能在NSCLC的多药耐药中有一定作用，并且有可能作为判断NSCLC恶性程度以及不良预后的生物学指标之一。

本研究通过检测SLC22A18在NSCLC中的表达为临床普遍存在腺癌比鳞癌更耐药并且耐药程度往往与肿瘤恶性程度相关的现象提供了理论根据，为进一步研究SLC22A18与NSCLC多药耐药的相关性提供了实验基础。我们有理由相信在NSCLC综合治疗尤其是化疗个性化用药时，SLC22A18可能成为一个重要的分子标志物。
